# Beyond Wishful Thinking: Integrating Consumer Preferences in the Assessment of Dietary Recommendations

**DOI:** 10.1371/journal.pone.0158453

**Published:** 2016-06-30

**Authors:** Xavier Irz, Pascal Leroy, Vincent Réquillart, Louis-Georges Soler

**Affiliations:** 1 Natural Resources Institute Finland (Luke), Economics & Society Research Unit, Helsinki, Finland; 2 Institut National de la Recherche Agronomique, INRA-ALISS, UR 1303, Ivry-sur-Seine, France; 3 Toulouse School of Economics, Université Toulouse Capitole (INRA), Toulouse, France; College of Tropical Agriculture and Human Resources, University of Hawaii, UNITED STATES

## Abstract

Convenience, taste, and prices are the main determinants of food choices. Complying with dietary recommendations therefore imposes a “taste cost” on consumers, potentially hindering adoption of those recommendations. The study presents and applies a new methodology, based on economic theory, to quantify this taste cost and assess the health and welfare effects of different dietary recommendations. Then, by comparison of those effects, we identify socially desirable recommendations that are most compatible with consumer preferences (i.e., that best balance health benefits against”taste cost”) and should be prioritized for promotion. The methodology proceeds in three-steps: first, an economic-behavioral model simulates how whole diets would change if consumers complied with dietary recommendations; second, an epidemiological model estimates the number of deaths avoided (DA) due to the dietary change; third, an efficiency analysis weighs the health benefits against the taste and policy costs of each recommendation. The empirical model is calibrated using French data. We find that **r**ecommendations to reduce consumption of red meat and soft-drinks, or raise consumption of milk products and fish/seafood impose relatively moderate taste costs. By comparison, recommendations related to F&V consumption and, to a lesser extent, butter/cream/cheese, snacks, and all meats impose larger taste costs on consumers. The F&V recommendation is the costliest for consumers to comply with, but it also reduces diet-related mortality the most, so that a large budget could be allocated to promoting F&V consumption while keeping this policy cost-beneficial. We conclude that promotion of most dietary recommendations improves social welfare. Our framework complements the programming models available in nutrition and public health: those models are best used to identify dietary targets, following which our framework identifies cost-beneficial ways of moving towards those targets.

## Introduction

Diet modeling has been widely used in the last decades to assess nutritional recommendations and dietary guidelines. Mainly based on Linear Programming (LP), those models aim at characterizing optimal diets optimizing an objective function (e.g., minimizing diet cost) subject to a set of constraints (e.g., nutritional requirements). The main rationale for the use of such models is that they help solve complex problems that arise because individuals need nutrients but eat foods, and, as nutrients are not evenly distributed in foods, there exists a large variety of possible dietary patterns compatible with a given set of nutritional requirements [1].

LP models have been used for different purposes in nutrition and public health [1]: assessment of the difficulty of complying with and compatibility of various nutrient- and food-based recommendations [2–5]; characterization of least-cost diets meeting a list of nutritional requirements [6]; translation of nutrient recommendations into food plans [7].

An important drawback of LP models, however, is that they may produce unrealistic diets as they fail to capture consumers’ preferences [8]. Hence, Henson [6] for the UK and Conforti and D’Amicis [9] for Italy found that it was possible to compose healthy diets which cost only 20 to 30% of observed cost and were composed of a very small number of food items. These results imply that food choices are not driven solely, or even mainly, by the satisfaction of nutritional needs [10,11] but that many other considerations come into play. Thus, models based only on nutritional aspects may produce diets that are incompatible with consumer preferences, which obviously cover many other characteristics of food products and diets.

These problems have been recognized in the research literature and partially addressed through the addition of palatability and social acceptability constraints. A real progress has been to consider, as a starting point of the analysis, the self-selected diets observed in representative samples of the population, and use objective functions such as the minimization of departure from those currently observed dietary patterns [12–16]. This approach relies on the idea that observed food choices reveal consumer preferences and the underlying trade-offs, for instance between palatability and costs, which influence consumers’ decisions [17]. Preferences changing only slowly, large departure from observed diets seem unrealistic [14].

However, even within this improved framework, the objective function of the programming problem remains arbitrary, which implies that the substitution possibilities among foods are exogenously defined by the modeler and neither theoretically nor empirically grounded. Thus, we argue that the programming models used to date in nutrition and public health to assess dietary guidelines do not integrate consumer preferences in a satisfactory manner, and that such a limitation has important consequences. First, there is little reason to believe that the substitutions among foods simulated by those models (for instance, the change in whole diet induced by a rise in fruits and vegetables (F&V) consumption) reflect the behavioral adjustments that would be made by”real” consumers. Second, LP models do not allow estimation of the full cost of dietary changes induced by the adoption of recommendations. This cost is not only financial, i.e. due to the change in expenditure to adopt the optimized diet, as it also includes the loss of well-being (or utility) created, at least in the short term, by the decision to comply with new dietary guidelines by consuming less preferred foods (”I decide to eat more broccoli to comply with the ‘5-a-day‘ rule, although I do not like broccoli much”). This short-term loss of hedonic rewards from a dietary adjustment is henceforth referred to as a taste cost. Third, as they do not permit calculation of taste costs, LP models cannot support the normative analysis of dietary recommendations, for instance by applying the cost-benefit or cost-effectiveness techniques widely used to assess the social desirability of treatments, drugs and other components of health care systems. This prevents a ranking of alternative recommendations, as well as an assessment of the social desirability of investing more or less in their promotion.

Overcoming those limitations appears to be a relevant challenge for nutrition and public health. Many studies show that standard dietary recommendations, for instance related to F&V, soft drinks, or snacks, are poorly adopted in many countries, especially among disadvantaged and less educated people [18–21]. This low level of compliance with nutritional recommendations might be explained by food prices and budgetary constraints [22], or nutrition knowledge [23], but we argue that it is also, and perhaps mainly, due to the cost that compliance imposes on consumers in terms of taste and convenience.

Hence, the goal of this article is to propose a new modeling approach to quantify this taste cost and identify dietary recommendations compatible, as much as possible, with consumers’ preferences. In effect, our contribution addresses, at least partially, the challenge recently formulated by Webb and Byrd-Bredbeener [11] to overcome consumer inertia to dietary guidance by “giv[ing] consumers control with nutrition messages that are realistic, positive, easy to understand, and actionable without an expectation that consumers will surrender foods they love”. Our diet modeling method is based on the economic theory of the consumer and parameterized using micro-level data on real food purchases in France. As it is paramount to propose simple messages to consumers, we simulate and compare the impacts of different food-based recommendations. Finally, we perform a cost-benefit analysis to establish how different recommendations should be prioritized.

## Methods and Data

The formulation and promotion of dietary recommendations remains the most popular policy instrument to induce consumers to make healthier food choices [24]. Yet the effect of such recommendations and their effect on social welfare is difficult to identify ex-ante. Our research seeks to develop and apply a tool to fill this knowledge gap [25]. To do so, we have developed a three-step methodology:

1. An economic-behavioral model predicts how whole diets would change if consumers complied with a given recommendation; for example to consume more F&V.
2. An epidemiological model estimates the number of deaths avoided (DA) due to the dietary change.
3. A cost-benefit assessment of the recommendation is carried out by balancing the taste cost of compliance with the recommendation and the policy cost of inducing consumers to change their behaviours against the monetized value of the health benefit from improved diets.

### The economic model of diet choice

The standard economic theory of consumer behaviour assumes that an individual chooses the amounts of goods she is going to consume in order to maximise a function–named”utility”—subject to a budget constraint. The utility function describes the preferences of a consumer which, in the case of food choices, relate to the taste of the goods, their convenience, and many other attributes. The budget constraint takes into account the prices of goods and available income. This optimisation program is referred to as the”nutritionally unconstrained problem”. Its solution, in the case of food choices, defines which goods are eaten and in which quantities. In this context, the adoption of a nutritional recommendation, such as eating a minimum quantity of F&V per day, is conceptualized as the integration of an additional constraint in the previous program. The additional constraint leads the consumer to modify her choices in order to comply with this new constraint and thus choose a modified set of goods (or the same goods but in different quantities). We call this new optimisation program the”nutritionally constrained problem”. Comparison of the solutions of those two programs provides two key results:

- First, the impact of the adoption of a nutritional recommendation on the entire diet, and hence a full characterization of the substitutions among foods that the recommendation has induced.
- Second, an estimate of the loss of utility, or taste cost, that the consumer incurred in the short term by adopting the nutritional recommendation. Adoption always reduces utility of the consumer because, if it was not the case, the consumer would have complied with the recommendation in the unconstrained situation.

An empirical difficulty arises because the utility function is not observed. However, assuming rational behavior as is standard in most analyses of consumer choices, observed consumption, given market prices and income, is just the solution of the”nutritionally unconstrained program”. This property is used to infer preferences from actual consumption data. Once the utility has thus been revealed and summarized in the form of price and income elasticities, economic theory is used to determine how a nutritional constraint affects choices and utility. To get the intuition of how the model works, let’s imagine a change in prices. We know that the rational consumer would adjust her consumption as a result of those price variations, and that this response is empirically quantifiable. When we simulate changes in consumption induced by compliance with a nutritional recommendation, prices are held constant, but we fall back on the economic framework by introducing “shadow prices”, defined as the set of prices that would have to prevail for the nutritionally-unconstrained individual to choose the exact same bundle of goods as the nutritionally-constrained individual. In other words, if the shadow prices were the current market prices, the consumer would spontaneously conform to the nutritional recommendation. For instance, the shadow price of red meat for the recommendation to decrease red meat consumption by 5% is the increase in the price of red meat that would be necessary for consumers to adopt the recommendation, all else being held constant. The difference between market price and shadow price would in that case be negative and akin to a price tax that would discourage consumption of red meat. Moreover, the shadow price differs from the market price for any food item which contains red meat (e.g. ready meals). However, because consumers substitute foods with one another, the optimal solution that minimizes the utility cost of complying with the recommendation also involves changes in all the foods that may substitute or complement red meat consumption. The economic theory then generates important insights:

- For any given food, the wedge between shadow price and market price is proportional to the per-unit nutritional contents (e.g. contents in F&V) of the goods appearing in the nutritional constraint (e.g. an increase in F&V consumption), and depends on demand elasticities. Shadow prices are thus empirically computable.
- From the set of shadow prices, we can deduce the change in consumption for each good. In particular, compliance with a nutritional constraint induces broad changes in the diet, even for those goods that do not appear directly in the constraint, because of relationships of substitutability and/or complementarity with the goods entering the constraint. That is to say that, for example, meat consumption might be affected by a recommendation to increase F&V consumption. This relationship of substitutability or complementarity between goods is expressed by the cross price elasticities of demand in the initial unconstrained situation. For example, the cross price elasticity of meat demand with respect to the price of fruits measures the responsiveness of meat demand to a change in the price of fruits. Hence, if we are able to estimate the price elasticities describing the behavior of the unconstrained individual, it is possible to infer the dietary adjustments resulting from compliance with the nutritional recommendation. We note that such elasticities, based on econometric analyses of food demand, are often used to assess the impacts of a nutritional tax on food choices [25]. Compared to these approaches developed to assess the effect of price variations on consumption and nutrient intakes, in this paper we consider the dual problem which consists of determining the price system and the compensation value such that a nutritional recommendation can be adopted without loss of utility.
- The taste cost of complying with a nutritional constraint is measured by the”compensating variation” (CV) of the dietary adjustment, defined as the amount of additional money a household would need to reach its initial utility after complying with the recommendation. The CV is calculated as the difference between observed expenditure (the food budget in the unconstrained situation) and the corresponding expenditure (i.e., food budget) that would be necessary to hold utility to its initial level when the nutrition constraint is imposed. This taste cost should be interpreted as a measure of the short-term loss of utility of the consumer which is a way to evaluate how costly/difficult it is to deviate from the unconstrained situation, ignoring the long-term health benefits of compliance, which are considered separately in the analysis (i.e., their measurement in terms of deaths avoided is presented in the next section and their valuation is discussed in the “Cost-benefit” section).

The theoretical and mathematical backgrounds of the model are presented in greater detail elsewhere [25].

### Health impact assessment

The health effects of consumption changes induced by the adoption of a nutritional recommendation are assessed with the DIETRON model. The DIETRON model provides estimates of the number of deaths avoided due to diet-related chronic diseases. As explained by Scarborough et al. ([26], p. 711):”the DIETRON model uses age- and sex-specific estimates of relative risk drawn from meta-analyses of trials, cohort studies and case–control studies, to estimate the impact on chronic disease mortality of counter factual population dietary scenarios”. The inputs of the DIETRON model are changes in intakes of the following foods and nutrient: fruits, vegetables, fibers, total fat, monounsaturated fatty acids (MUFA), polyunsaturated fatty acids (PUFA), saturated fatty acids (SFA), cholesterol, salt, and energy. The exact pathways to specific diseases (e.g., stroke) and intermediate risk factors are described in the paper that first presented the model [27]. The studies covered by the meta-analysis of risk factors are clearly listed in Table 1 of the same reference, while the relative risk ratios used in DIETRON are published in Table A2 of the Appendix of reference [26].

**Table 1:** Changes in food consumption induced by the imposition of dietary constraints (right percentage in each column) and baseline contribution of each food group to the constrained food (left percentage in each column) for the “Lower-average” consumer type.*

	F&V +5%		Red meat -5%		All meats -5%		Salty/Sweet fat prod. -5%		Soft drinks -5%		Milk prod. +5%		Butter, cream & cheese -5%		Fish & seafood +5%	
	%	%	%	%	%	%	%	%	%	%	%	%	%	%	%	%
Red meat	0.0	-9.1	89.7	-5.5	22.7	-8.2	0.0	2.1	0.0	0.3	0.0	-1.5	0.0	0.6	0.0	-0.9
Other meats	0.0	6.2	0.0	0.7	38.8	-6.4	0.0	3.4	0.0	0.4	0.0	0.1	0.0	7.0	0.0	-0.1
Cooked meats	0.0	-3.3	0.0	0.8	32.2	-1.3	0.0	-2.4	0.0	-0.1	0.0	-1.1	0.0	0.8	0.0	-0.2
*Meat aggregate*	*0*.*0*	*-0*.*3*	*89*.*7*	*-0*.*7*	*93*.*7*	*-5*.*2*	*0*.*0*	*1*.*2*	*0*.*0*	*0*.*2*	*0*.*0*	*-0*.*6*	*0*.*0*	*3*.*6*	*0*.*0*	*-0*.*3*
Milk products	0.0	-4.3	0.0	0.7	0.0	3.3	0.0	1.2	0.0	0.5	100.0	5.0	0.0	-0.9	0.0	0.0
Cheeses, butters, fresh creams	0.0	-2.9	0.0	0.1	0.0	4.2	0.0	-4.8	0.0	-0.1	0.0	0.2	100.0	-5.0	0.0	0.1
*Dairy pdts*	*0*.*0*	*-4*.*0*	*0*.*0*	*0*.*6*	*0*.*0*	*3*.*4*	*0*.*0*	*0*.*0*	*0*.*0*	*0*.*3*	*100*.*0*	*4*.*1*	*100*.*0*	*-1*.*7*	*0*.*0*	*0*.*0*
Fish	0.0	9.7	0.0	1.7	0.0	7.5	0.0	2.4	0.0	0.1	0.0	0.2	0.0	-0.8	96.1	5.3
Eggs	0.0	-7.6	0.0	-0.8	0.0	-3.3	0.0	1.3	0.0	0.5	0.0	-0.7	0.0	-9.8	0.0	-1.0
Animal products	*0*.*0*	*-2*.*3*	*89*.*7*	*0*.*3*	*93*.*7*	*1*.*1*	*0*.*0*	*0*.*5*	*0*.*0*	*0*.*3*	*0*.*0*	*2*.*4*	*0*.*0*	*-0*.*5*	*96*.*1*	*0*.*2*
Grains	0.2	-6.2	0.0	-1.0	0.0	-0.3	0.0	-3.2	0.0	-0.2	0.0	-2.3	0.0	-3.2	0.0	-1.4
Potatoes	0.0	-27.6	0.0	-0.8	0.0	-4.5	0.0	4.5	0.0	-1.5	0.0	-1.2	0.0	8.5	0.0	-1.0
Starchy food	*0*.*2*	*-16*.*1*	*0*.*0*	*-0*.*9*	*0*.*0*	*-2*.*2*	*0*.*0*	*0*.*4*	*0*.*0*	*-0*.*8*	*0*.*0*	*-1*.*8*	*0*.*0*	*2*.*2*	*0*.*0*	*-1*.*2*
Fruits—Fresh	40.7	-1.1	0.0	1.5	0.0	2.7	0.0	-1.8	0.0	0.3	0.0	-0.4	0.0	-3.3	0.0	1.0
Fruits—Processed	2.8	27.0	0.0	0.2	0.0	-3.2	0.0	3.6	0.0	1.0	0.0	-5.2	0.0	-8.3	0.0	-0.5
F&V juices	6.3	4.0	0.0	0.8	0.0	-0.3	0.0	7.9	0.0	0.8	0.0	-1.8	0.0	-0.6	0.0	0.4
Vegetables—Fresh	32.6	9.5	0.0	-0.5	0.0	-0.3	0.0	0.0	0.0	0.1	0.0	0.4	0.0	6.7	0.0	0.0
Vegetables—Processed	9.9	18.4	0.0	0.0	0.0	-2.7	0.0	1.5	0.0	0.5	0.0	-1.6	0.0	4.4	0.0	-0.4
Fruits—Dry	0.5	-6.0	0.0	1.4	0.0	11.7	0.0	2.7	0.0	-1.7	0.0	6.6	0.0	-2.5	0.0	1.5
F&V aggregate	*92*.*7*	*5*.*9*	*0*.*0*	*0*.*5*	*0*.*0*	*0*.*8*	*0*.*0*	*-0*.*5*	*0*.*0*	*0*.*3*	*0*.*0*	*-0*.*3*	*0*.*0*	*1*.*1*	*0*.*0*	*0*.*4*
Ready meals	4.2	-11.7	10.1	-1.1	6.3	-3.6	0.0	-1.6	0.0	-0.6	0.0	-1.3	0.0	1.4	3.8	-2.9
Oil, margarine, condiments	0.0	12.0	0.0	0.1	0.0	-1.2	0.0	11.1	0.0	0.4	0.0	1.0	0.0	9.3	0.0	0.1
Salt-fat products	0.0	-20.7	0.1	1.2	0.1	10.3	8.1	-8.5	0.0	-1.4	0.0	-2.7	0.0	-28.5	0.0	-0.3
Sugar-fat products	2.9	2.1	0.0	0.1	0.0	0.3	91.9	-4.7	0.0	0.2	0.0	-0.2	0.0	-2.7	0.0	-0.2
Soft drinks	0.0	-18.4	0.0	0.7	0.0	5.3	0.0	2.8	100.0	-5.0	0.0	-3.5	0.0	-3.2	0.0	-0.1
Water	0.0	-20.0	0.0	1.8	0.0	10.0	0.0	-0.7	0.0	-0.2	0.0	-0.2	0.0	3.0	0.0	0.1
Alcoholic beverages	0.0	12.9	0.0	0.3	0.0	-0.4	0.0	3.7	0.0	-0.2	0.0	0.2	0.0	1.6	0.0	-0.5

* Food groups are defined as in [29]: Red meat (beef and veal); other meats (poultry, pork, lamb, etc.); cooked meats (ham, pâté, sausages, bacon, etc.); milk products (milk, yoghurt, dairy desserts, etc.); cheese, butter and cream; fish and seafood; eggs; grain products (bread, pasta, rice, wheat flour, and cereals); potatoes; fresh fruits; processed fruits (canned & compote); fruits & vegetables juices; fresh vegetables including legumes; processed vegetables including legumes (canned, frozen); dried fruits & nuts; ready meals (pizza, sauerkraut, cassoulet, etc.); oils & vegetable fat; salt-fat products (finger food, chips, crackers, appetizers); sugar-fat products (candy, chocolate, cookies, pastry, ice cream, jam, etc.); soft drinks (sodas, lemonade, syrups, etc.); mineral and spring waters; alcoholic beverages.

The output of the economic model is a vector of changes in consumption of goods when the consumer conforms to a nutritional recommendation. To link it to DIETRON, we use a composition table that provides for each good its content in the different foods and nutrients entering the DIETRON model.

### Cost-benefit analysis

The overall effect of each recommendation on social welfare is established by comparing the monetary value of its costs and benefits. In this framework, the expressions”welfare improving” and “cost-beneficial” refer to situations where the health benefits outweigh the costs as defined in this section. The long-term health benefit from compliance with a recommendation is monetized by multiplying the number of DA, calculated from the health impact assessment outlined in the previous section, by the value of a statistical life, which is interpreted as the effort, in terms of the resources used, that society is willing to make in order to reduce the risk of death [28]. On the cost side, the short-term taste cost imposed on consumers, which was discussed above in the presentation of the economic model, represents a first component. However, we also need to include the costs of implementation of policies and interventions necessary to bring about compliance with each recommendation. This is important because, while the model calculates the effects of dietary adjustments under an “as if” assumption, i.e. assuming compliance with the nutritional recommendation, in practice behavioural change requires public investment in social marketing campaigns and other types of interventions.

A problem arises, however, because quantifying the cost of a policy inducing consumers to change their consumption of a given food or nutrient by a certain amount is very difficult. We circumvent the issue by defining a cost-benefit threshold characterizing the maximum amount that could be invested to promote a given recommendation while ensuring that the benefits outweigh the costs. This is achieved by balancing the health benefits generated by a nutritional recommendation (denoted *Bh)* against the cost to individuals (denoted *CC* and measured by the short-term taste costs) and the cost of public sector interventions (*C*_*P*_). The cost-benefit threshold is then simply calculated as *C*_*P*_ *= Bh–CC*.

### Empirical assessment

The empirical implementation of the economic model requires different sets of data. To determine the initial consumption of foods and the economic parameters (elasticities), we use the source of data and results of the most recent available econometric analysis of food demand in France conducted by Allais et al. [29]. That study is based on data from a representative panel of French households (KANTAR Worldpanel) over a five-year period from 1996 to 2001. The participating households record weekly all their purchases of foods, using bar code scanning technology whenever possible, but foods without bar codes are also recorded. The information provided includes the characteristics of the purchased product (e.g., brand, size), the quantity purchased as well as related expenditure. KANTAR also provides the main socio-economic characteristics of the panel households, including household size, region of residence and income class. Each annual round of the data set contains information on approximately 5,000 households, with an annual rotation of roughly one third of the participants.

For the sake of consistency, we had to use the same 22 product aggregates as study [29] from which the elasticities are drawn. Those elasticities in [29] are reported for four representative types of households differentiated by income quartiles and labeled in what follows as “Modest”, “Lower average”, “Upper average”, and “Well-off”. The nutrient contents of the 22 food aggregates are calculated by combining the food composition database of the French dietary intake survey INCA2 and average adult intakes of the component foods of each aggregate drawn from INCA2 (which stands for “Étude Individuelle Nationale des Consommations Alimentaires 2006–7”), which are freely available from the open data platform of the French government (https://www.data.gouv.fr/fr/datasets/donnees-de-consommations-et-habitudes-alimentaires-de-letude-inca-2-3/). The model is applied to estimate the variations in households’ purchases induced by the adoption of eight food-based recommendations taken one at a time. We provide some details about the choice of recommendations at the beginning of the results section. The effects of dietary adjustments are calculated under an “as if” assumption, i.e. assuming that the households comply with a 5% change in the constraint level (for example in the case of F&V, we assume that a consumer has to increase his consumption of F&V by 5%).

To simulate the health effects, changes in food purchases at household level, as calculated by the economic model, are translated into changes in individual intakes, distinguishing between males and females and using the INCA2 dietary intake database. This is accomplished under the assumption that (i) all household members experience the same relative changes in intakes, and (ii) the relative changes in consumption of food-at-home and food-away-from-home are equal. Variations in nutrient intakes are then calculated from variations in food intakes by using the nutritional coefficients of the 22 aggregates. Finally, the changes in nutrients are fed into DIETRON model so as to estimate the health effects of the dietary adjustments.

The parameters of the DIETRON model are derived from world-wide meta-analyses of dietary risk factors and are not country specific, so that adapting the DIETRON model to France only requires calibration of the initial mortality levels, by relevant causes. This is achieved by using the INSERM data on mortality in France attributable to major diet-related diseases (INSERM stands for “Institut National de la Santé et de la recherche Médicale”). We limit the study to individuals between the age of 25 and 74 and therefore focus on the effects of dietary changes on premature deaths. The chronic diseases considered in DIETRON account for slightly more than one third of total French mortality.

Finally, the health benefit is quantified by applying a monetary value to the reduced mortality figures calculated by DIETRON. Estimates of the value of a statistical life vary substantially across countries and policy domains. In the following analysis, we use a conservative value of a statistical life, based on the cost threshold of a Quality Adjusted Life Year that is applied in the UK to investigate the cost-effectiveness of medical care (e.g., drugs, procedures). As explained in Irz et al. [25] this provides a value ranging from €240k to €360k.

In addition to the mean value of the different relative risks, the DIETRON model also provides the distributions of each relative risk parameter. Twenty-six of those follow log-normal distributions and six follow normal distributions, while all are independent. We perform Monte Carlo simulations by drawing a set of parameters one million times. To be more precise, for a given type of consumer, we draw a set of 32 parameters, use this set to evaluate the number of DA for each nutritional recommendation and repeat the operation one million times. In the results section, we provide the median as well as the 2.5 and 97.5 percentiles of DA for each recommendation.

The R codes to simulate both the economic model and the epidemiological model are available upon request from the authors.

## Results

Comparison of our data describing the average diet of French consumers with conventional dietary norms confirms previous findings that the French diet is rich in fat, particularly of the saturated kind that originates primarily from animal products [14,15]. This justifies the analysis of three food-based recommendations aimed at reducing consumption of: all meats; red meat, because of its particularly high content in saturated fatty acid (SFA); and SFA-rich dairy products, which include butter, cream, and cheese. On average, intake of calcium in the French population complies with dietary reference intake, but is too low for some consumer groups (mainly women and the low-income). This shortcoming could be addressed by raising consumption of the other dairy products (henceforth referred to as “milk products”). Snacking has also been identified in France as a potentially growing issue, particularly among young consumers [30], and we therefore consider recommendations to reduce consumption of both salty and sweet snacks (henceforth referred to as “salty/sweet-fat products”). Sugar-sweetened beverages are increasingly recognized as an important determinant of body weight [31] and a recommendation to reduce their consumption is appropriate in France given concerns over obesity and overweight in the population. Consumption of F&V falls well below the recommended five portions a day, and this is particularly true for low-income consumers. A F&V recommendation is therefore included. Finally, there is moderate but consistent evidence regarding the health benefits of increased fish consumption [32], and we therefore analyse the impact of a related recommendation.

Table 1 presents the simulated dietary adjustments for the “lower average” household type. Although results for the other types are not reported here, we have found that they are broadly similar to those presented below. This similarity in the way the four household types respond to recommendations is explained by small differences in the price and income elasticities of those household types. Those elasticities, which we used to calibrate the model, are reported in Tables 6–10 of the supplementary material of reference [29]. For each constraint, presented in a separate column, Table 1 depicts first the baseline contribution of each food group to the constrained food, then the consumption changes that would result from compliance with the constraint.

Turning to the changes in diet and focusing first on the”all meats” recommendation, we note that the five percent decrease that is imposed exogenously corresponds to an absolute decline of roughly eight grams per day. This quantitatively small reduction in meat consumption triggers relatively important dietary adjustments. Starchy products are complements of meats and thus their consumption decreases with the reduction in meat consumption (-2.2%). On the contrary, dairy products, which provide proteins, are meat substitutes and thus their consumption increases with the exogenously-imposed reduction in meat consumption (+3.4%). Looking at the results at a lower level of product aggregation reveals that complex adjustments also take place within broad food categories. Thus, consumers choose to compensate the decrease in meat consumption by raising consumption of fish (+7.5%) but also, and less expectedly, by reducing that of eggs (-3.3%).

To avoid confusion in interpreting the results, we note that the decrease in consumption of the “Meat aggregate” category is different from 5% (the target for a decrease in total consumption of meat). This is because a food category (ready meals) also contains some meat. Then, the change in “All meats” consumption takes into account the changes in consumption of the “Meat aggregate” as well as the changes in the consumption of the other food categories which contain some meat. This remark applies to other recommendations (e.g., on F&V).

The dietary adjustments induced by compliance with the constraints are heterogeneous. Compared to simulated adjustments for the”all meats” constraint, imposition of the constraint on red meat produces smaller consumption changes. This is understandable as this constraint is less demanding in the sense that it restricts a smaller fraction of the diet (the decrease in red meat consumption is about 3 g/day) and substitution with other meats occurs leading to a small decrease in aggregate meat consumption (-0.7%). Overall, the “red meat” constraint affects consumption of the different food groups in the same direction as, but with a lower magnitude than, the “all meat” constraint.

The recommendation to increase consumption of F&V by 5% induces the largest adjustments in the diet. Thus, consumption of starchy products (-16.1%), dairy products (-4.0%), ready meals (-11.7%), oil and margarine (+12.0%), and salt-fat products (-20.7%) are strongly affected. Within the F&V category, and in terms of absolute quantity, the biggest increase is for fresh vegetables whereas fresh fruit consumption decreases by a very small amount. However, the largest percentage increases are for processed products, meaning that the adoption of the recommendation raises the relative importance of processed products within the F&V category. Regarding the other recommendations, it is worth noting that an increase in consumption of milk products leads to a decrease in consumption of meat (particularly red meat), starchy products, and salt-fat products. A decrease in consumption of butter-cream and cheese induces an increase in consumption of meat, particularly “other meats”, oil and margarine, and starchy products; it also results in a (large) decrease in the consumption of salt-fat products. An increase in fish consumption leads to a decrease in consumption of ready meals, starchy foods, and red meat. A decline in consumption of salt/sweet-fat products leads to an increase in oil and margarine consumption and a decrease in consumption of cream-butter and cheese. Finally, a decrease in consumption of soft drinks has a relatively small impact on consumption of the different food products.

Next, variations in consumption for the four consumer types are translated into changes in intakes, and Table 2 summarizes the results for the whole population. Complex adjustments in the nutrient profile of the diet occur, and the overall effect on diet quality is ambiguous. Thus, in most case there are nutritionally beneficial changes but also adverse ones. For most recommendations, positive changes in the diet relate to lower intakes of salt and energy intake, and a decrease in energy density. Adverse effects, however, are also evident. For example, for all recommendations, fiber intake decreases and total fat intake rises. Five recommendations (on”all meats”,”red meat”,”soft drinks”,”milk products”, and”fish and seafood”) have relatively small impacts on nutrient intakes, i.e. lower than one percent (in absolute value) for most nutrients. Recommendations on the consumption of”salty/sweet-fat products”, and”butter-cream and cheese”, have larger impacts, as the changes in the intake of some nutrients are larger than one percent (in absolute value). The recommendation on F&V induces much larger changes, often in excess of two percent (in absolute value). It is worth noting large reductions in intakes of energy, salt and carbohydrates, and that some changes seem counter-intuitive. For instance, the increase in F&V intake leads to a decrease in fiber intake. This is caused by multiple substitutions within the diet, especially the reduction in consumption of grains, potatoes and ready meals induced by compliance with the F&V constraint. We can also note a reduction in iron intake mainly due to a reduction in consumption of grains, potatoes, meat, ready meals; a reduction in potassium intake attributable to a decrease in consumption of potatoes, ready meals and dairy products; and a reduction in calcium intake caused by a drop in consumption of dairy products, ready meals and water.

**Table 2:** Change in the nutritional profile of the diet (whole population).

	F&V +5%	Red meat -5%	All meats -5%	Salty/Sweet fat prod. -5%	Soft drinks -5%	Milk prod. +5%	Butter, cream & cheese -5%	Fish & seafood +5%
	Percentage variations (%) in nutritional factor/indicator							
**DIETRON nutritional factors (units)**								
Fruits (g)	1.73	1.11	1.84	-0.85	0.36	-0.76	-3.20	0.67
Vegetables (g)	7.04	-0.47	-1.29	-0.03	0.07	-0.19	5.39	-0.54
Fibers (g)	-2.32	-0.24	-0.33	-1.86	-0.13	-0.98	-0.36	-0.67
Total Fat (% energy)	1.40	0.11	0.12	1.47	0.10	0.52	1.04	0.21
MUFA (% energy)	2.57	0.10	-0.32	3.13	0.15	0.58	2.25	0.22
PUFA (% energy)	4.66	0.23	-0.06	5.09	0.12	0.61	3.06	0.41
SFA (% energy)	-0.35	0.11	0.85	-1.20	0.07	0.54	-0.81	0.19
Cholesterol (% energy)	-0.82	-0.09	-1.00	-0.46	0.18	0.18	-1.22	0.20
Salt (g)	-5.13	-0.23	0.30	-2.84	-0.23	-1.06	-2.65	-0.78
Energy (MJ)	-2.35	-0.23	-0.30	-0.85	-0.10	-0.49	-0.76	-0.55
**Other nutritional indicators (units)**								
Energetic density (kcal/100g)	-0.76	-0.13	-0.13	-0.21	-0.02	-0.17	-0.71	-0.16
Proteins (g)	-3.06	-0.46	-1.14	-0.87	0.00	-0.32	-0.06	-0.34
Available carbohydrates (g)	-4.49	-0.29	-0.11	-2.40	-0.22	-1.06	-2.01	-0.81
Lipids (g)	-1.03	-0.12	-0.16	0.59	0.00	0.03	0.26	-0.33
Ca (mg)	-4.92	0.23	2.42	-1.93	0.00	0.81	-1.25	-0.28
Fe (mg)	-2.08	-0.34	-0.72	-1.00	-0.06	-0.69	-0.04	-0.52
K (mg)	-3.56	-0.01	0.09	0.15	-0.07	-0.10	1.04	-0.24

Regarding the public health impacts, it is reassuring that every nutritional recommendation reduces the mortality due to chronic diseases. Table 3 displays the results of Monte-Carlo simulations in terms of DA. There is some correlation between the relative magnitude of variations in nutrient intakes and the number of DA. Thus, the recommendation on F&V, which induces the largest changes in nutrient intakes, has by far the largest impact on mortality, as it might save as much as 2507 (2228–2790) DA per annum, which is about 3.8% of the total number of deaths taken into account in the DIETRON model. Recommendations on”salty/sweet-fat products” and”butter-cream-cheese” have intermediate impacts on the number of DA (569 (479–662) and 696 (551–838), respectively), although it would have been difficult to rank the health impacts of those two constraints on the sole basis of the changes in nutrient intakes. Finally, the other recommendations save less than 400 DA (median value). Among those, the recommendation on”fish and seafood” has the largest impact and the recommendation on soft drinks the smallest. It should be acknowledged that the rather small impact on health of lowering soft drink consumption might be explained by the relatively small average consumption in the French adult population. For instance, a recent study [33] reported that sales of sugar-sweetened beverages in France were among the lowest among EU countries, at about 52 calories sold per capita per days, as compared to almost twice that amount in Germany and more than three times that level in the USA. This example highlights the need to use caution when generalizing our conclusions to other countries because differences in country-specific contexts matter to health outcomes, as claimed with reference to taxation of soft drinks by other authors [34].

**Table 3:** Health effects of the simulated dietary adjustments (DA: deaths avoided).

	F&V +5%	Red meat -5%	All meats -5%	Salty/Sweet fat prod. -5%	Soft drinks -5%	Milk prod. +5%	Butter, cream & cheese -5%	Fish & seafood +5%
DA for DIETRON diseases	2,507	230	245	569	118	251	696	395
(95% confidence interval)	(2228–2790)	(198–261)	(193–298)	(479–662)	(106–131)	(199–305)	(551–838)	(342–449)
% CHD	34	28	21	44	28	36	44	35
% Stroke	17	19	22	21	19	26	12	20
% Cancers	49	53	57	35	53	38	44	45
Share (%) of mortality avoided								
Modest & Lower average	4.54	0.33	0.26	0.69	0.18	0.41	0.88	0.56
Upper average & Well-off	2.69	0.38	0.54	1.11	0.17	0.33	1.33	0.65

Conversely, the recommendation on fish and sea food has a surprisingly large effect if one takes into account the rather small change in consumption that is imposed (the 5% rise is worth 2g/day). It is partially due to the decrease in consumption of meat and ready meals.

It is also interesting to note that the two recommendations to limit meat consumption have about the same impact on health whereas the changes in nutrient intakes induced by a decrease in red meat consumption are much lower than those induced by the decrease in meat consumption. The main reason is that the”all meats” recommendation leads to a much stronger increase in consumption of”cheese, butter, cream” and salt-fat products.

On average, about 35% of DA is attributed to coronary heart disease (CHD), 20% to strokes and 45% to cancers. This is linked to the initial number of deaths due to each of the three groups of non-communicable diseases (NCD) considered here. Some recommendations have, however, a relatively larger impact on CHD (”salty/sweet-fat products”,”cream-butter and cheese”) whereas others have a relatively larger impact on cancers (”F&V”,”red meat”,”all meats”).

In order to investigate equity effects, Table 3 also displays the share of mortality avoided separately for low-income consumers, defined by an income less than the median, and high income consumers (defined symmetrically). It should be acknowledged that the prevalence of diet-related chronic diseases among the low-income is higher than that in higher income categories, which is explained in part by less healthy consumption patterns. A reduction in health inequity is, however, not achieved by all the recommendations. The F&V recommendation and, to a lesser extent, the milk products recommendation, induce a reduction in health inequity as the share of avoided mortality is relatively larger among the low income. The other recommendations increase health inequity, with the exception of the recommendation to reduce consumption of soft drinks, which is neutral from that point of view.

It may first seem surprising that while the relative dietary adjustments are largely similar across the four income groups, the health effects vary significantly. This is explained by the fact that, in addition to those dietary adjustments, the health effects depend also on the initial diets and initial mortality levels by type of diet-related diseases, which differ across the four income groups.

The taste costs borne by consumers are clearly linked to the magnitude of the dietary changes induced by the adoption of the recommendations and can be large (Table 4). Thus the 5% increase in F&V consumption imposes a high taste cost (€466 million) when compared to other recommendations. That recommendation has the largest health effect but is also costliest to consumers. By comparison, the red meat, soft drinks, milk products and fish recommendations deliver small numbers of DA at a very low taste cost. We can then oppose recommendations which have smaller health effects (in terms of DA) but are not costly to consumers (red meat, soft drinks, milk product and fish) and the F&V recommendation which has strong health effects but is costly to consumers. The”all meats” recommendation also appears very costly to consumers. Finally, recommendations on”salty/sweet-fat products” and”butter, cream, and cheese” both generate moderate health gains while imposing moderate taste costs.

**Table 4:** Health benefits and taste cost of the dietary adjustments (median and 2.5 and 97.5 percentiles derived from Monte Carlo simulations).

	F&V +5%	Red meat -5%	All meats -5%	Salty/Sweet fat prod. -5%	Soft drinks -5%	Milk prod. +5%	Butter, cream & cheese -5%	Fish & seafood +5%
Taste Cost (M€)	466	10	76	89	1	13	109	10
% food budget	0.64	0.01	0.10	0.11	0.00	0.02	0.14	0.01
DA	2,507	230	245	569	118	251	696	395
(95% confidence interval)	(2228–2790)	(198–261)	(193–298)	(479–662)	(106–131)	(199–305)	(551–838)	(342–449)
% DA (on DIETRON diseases)	3.8	0.3	0.4	0.9	0.2	0.4	1.1	0.6

Table 5 presents estimates of the cost-benefit threshold *C*_*p*_ defined as the maximum amount that could be invested to promote a recommendation while ensuring that the outcome remains cost-beneficial (i.e., the health benefits, measured by the monetary value of DA, outweigh the policy and taste costs). Using very conservative assumptions to value health, it turns out that, except for the”all meats” recommendation, this budget threshold is much larger than what is typically used to design and run information campaigns. For instance, Capacci and Mazzocchi [35] report that the ambitious “5-a-day” UK campaign to encourage consumption of F&V, which was partially successful since it raised consumption by 8%, had a total budget of less than £3 million (roughly €4 million). Thus, if one believes that a population-wide intervention achieves the target of 5% change in consumption, the conclusion follows that promotion of most healthy-diet recommendations is socially desirable. Further, the results also give a simple criterion to rank the different recommendations: on the basis of the budget threshold *C*_*p*_, healthy-eating messages targeting the consumption of F&V as well as fish/seafood should be prioritized. The recommendation on SFA-rich dairy products is likely to perform well too, but there is a larger uncertainty on its impact on health.

**Table 5:** Monetized benefits, costs and cost-benefit thresholds of the recommendations (1DA = €240k).

Food based recommendations	Health Benefits (M€)	(95% confidence interval)	Taste Cost (M€)	C_p_ Max Campaign (M€)	(95% confidence interval)
F&V +5%	602	(535–670)	466	136	(68–203)
Red meat -5%	55	(48–63)	10	45	(37–52)
All meats -5%	59	(46–72)	76	-17	(-30 - -5)
Salty/Sweet fat prod. -5%	137	(115–159)	89	48	(26–70)
Soft drinks -5%	28	(25–31)	1	27	(24–30)
Milk prod. +5%	60	(48–73)	13	47	(35–60)
Butter, cream & cheese -5%	167	(132–201)	109	58	(23–92)
Fish & seafood +5%	95	(82–108)	10	85	(72–98)

## Discussion

Recently, the US Dietary Guideline Advisory Committee (DGCA), seeking to assess the desirability of reformulating the 2010 guidelines, concluded that”the overall body of evidence identifies that a healthy dietary pattern is higher in vegetables, fruits, whole grain, low- and non-fat dairy, seafood, legumes, and nuts; lower in red and processed meat; and low in sugar-sweetened foods and drinks and refined grains” [36], Part A, p. 4). These recommendations express clear dietary targets but leave some questions open. Hence, given the current state of consumer preferences, what are the paths of least resistance for consumers to move towards those targets? In other words, which recommendations must be prioritized in order to deliver the largest health benefits while imposing the lowest policy and taste costs? Our analysis provides some important insights about those questions.

First, the simulations conducted on French data reveal that a rational consumer would respond to diet recommendations through large and complex changes in consumption, which implies that an accurate assessment of the health and economic effects of such recommendations requires a whole diet approach. The substitutions in the diet are functions of consumers’ preferences for foods, which cannot be established a priori but should be estimated on the basis of observed choices. Further, those adjustments may reinforce the impacts of some recommendations. For instance, the adoption of the F&V recommendation induces other nutritionally- favorable changes, such as a reduction in consumption of meat, ready meals and salt-fat products. However, compliance can also have adverse effects in some nutritional dimensions: the same F&V recommendation leads to reductions in consumption of milk products and intake of calcium. It also results in a reduction in fiber, potassium, and iron intakes. Even if the overall health impacts estimated with the DIETRON model are positive, such results must be taken into account in the design and communication of dietary recommendations, especially for sub-populations with higher risks of nutritional deficiencies. For instance, the decrease in calcium intake induced by the adoption of the F&V recommendation may be detrimental to some sub-groups of the population. Similarly, regarding iron and potassium, we know that intakes by French women are on average below recommended values. In this case, promotion of F&V consumption should ideally be accompanied with messages aimed at limiting the risk of worsening deficiencies in those nutrients (for instance, by not decreasing too much consumption of red meat or dairy products). This also suggests that further research is needed to identify combinations of different constraints best suited to avoid such negative consequences in specific sub-populations.

Second, food-based recommendations studied in this article differ widely in terms of both their health impacts and the taste costs that they impose on consumers. Recommendations to decrease by 5% consumption of red meat and soft drinks, or increase by 5% consumption of milk products and fish/seafood generate moderate taste costs. Conversely, recommendations related to F&V consumption and, to a lesser extent, butter/cream/cheese, salty-sweet fat products, and all meats create larger taste costs. Compliance with the F&V recommendation generates the largest taste-cost, but also, in terms of public health, the largest health benefits (the largest number of DA). The simulations indicate, however, that high budgets could be devoted to promoting F&V while keeping this policy cost-beneficial. More generally, our analysis suggests that allocating more resources to the promotion of several food-based recommendations would be welfare-improving in France.

From a methodological angle, the economic model presented here has two advantageous characteristics over the programming models used to date to investigate diets in nutrition and public health. First, it is based on the economic concept of preferences, which are revealed by consumers’ actual choices, and the response of those choices to exogenous changes (e.g., rise in price). This introduces realism when defining preferences and circumvents the difficult problem of imposing palatability and acceptability constraints in an ad-hoc and somewhat arbitrary manner. Second, the explicit consideration of preferences (i.e., utility) into the model makes it possible to estimate the tastecost of compliance with recommendations or, in other words, the reduction in hedonic and other rewards created by compliance with those recommendations.

Another contribution of our approach is to establish a clear link between the economic-behavioural model of food choice under dietary constraints and an epidemiological model quantifying the impact of dietary changes on mortality. This allows us to simulate, within a fully consistent framework, the effect of adoption of nutritional recommendations on short-run hedonic rewards and long-term health for several income groups of the French population. The analysis proceeds further by integrating health and welfare effects into a cost-benefit measure, which permits a ranking and comparison of recommendations.

Our analytical framework complements approaches previously developed in nutrition and public health and based on programming models. Those models can be used to identify dietary targets, following which the economic-behavioural model can be applied to identify cost-beneficial ways of moving towards those targets. However, we must also acknowledge shortcomings of our economic-behavioural approach. First, it is only suitable to assess the effect of marginal (i.e., small) changes. While this is not a problem to decide which types of diet recommendations should be promoted in a given country at a given time, which seems to be the most relevant policy question, the feature also means that we are not able to identify a unique optimal diet. Second, the simulations can only be performed at a relatively high level of product aggregations (i.e., in the range of 20 product groups). Third, as the price elasticties are estimated for consumer groups rather than individuals, they give information on average preferences, which is valid to address public health issues but unsuitable for individual counselling. For example, consider the case of a sub-population of individuals already consuming large amounts of F&V, much larger than the recommendation. First, from a nutritional point of view, as shown in this article, promoting further increase in F&V consumption might have adverse effects, by decreasing iron and potassium intakes, possibly to undesirably low levels. Second, this sub-population might have preferences which strongly differ from those of the average population, reflecting behavioural differences. Applying elasticities estimated for the whole population to this sub-population would then likely produce results and recommendations that are not consistent with their preferences.

We must also acknowledge that the validity of the estimated health benefits and associated policy recommendations depend heavily on the reliability of the DIETRON model. Although that model has been validated and conveys the main aspects of the prevailing consensus in nutritional epidemiology, the lively debate and lack of certainties in that scientific area should be kept in mind when interpreting our results. To illustrate with one example, although a reduction in saturated fat intake remains a key dietary objective in most countries, researchers have started questioning the scientific basis of that recommendation [37,38]. While such issues fall beyond the scope of this paper, they suggest the need to test the robustness of our approach and conclusions by using alternative epidemiological models. Further, the economic model is also subject to uncertainties, which we were unfortunately unable to address as we do not have information about the joint distribution of the elasticity parameters and an assumption of independence of the elasticities would be inconsistent with the theory of the consumer upon which the model is based.

Despite these limitations, the results demonstrate the policy relevance of the approach, showing clear differences in health benefits, taste costs, and benefit-cost balance across recommendations.

We close by pointing out other potential applications of the method to assess recommendations of a different nature, for instance nutrient-based recommendations (e.g., targeting salt), and sustainable recommendations taking into account the environmental impact of diets. It would also be possible to focus on specific nutritional issues by disaggregating food categories (e.g., fatty versus non-fatty fish, processed versus fresh F&V) or to investigate specific consumer clusters of interest. For instance, grouping consumers using criteria other than income would permit to differentiate the impact of recommendations according to age, education, or BMI, the only hard requirement for new applications being the availability of price elasticities congruent with the corresponding food groups and consumer categories. Our analytical framework could also be applied to investigate the impact of recommendations on individuals with healthy initial diets who could be at risk of consuming excessive amounts of foods or nutrients considered “healthy” for the population at large (e.g., F&V). Further research may also compare the benefit-cost balance of food and nutrient-based recommendations in different countries, since that balance depends on country-specific consumption patterns and consumer preferences (as captured by elasticities).
